# COVID‐19‐associated AMPA‐R and CRMP‐5 autoimmune encephalitis in a patient with thymoma and myasthenia gravis

**DOI:** 10.1002/ccr3.7064

**Published:** 2023-03-19

**Authors:** Jenelle Raynowska, Victoria Wu, Max Kazer, Jamie Nicole LaBuzetta, Dominic Ferrey, Anastasie Dunn‐Pirio

**Affiliations:** ^1^ Department of Neurosciences University of California, San Diego La Jolla California USA

**Keywords:** autoimmune encephalitis, COVID‐19, myasthenia gravis, thymoma

## Abstract

Thymomas are associated with autoimmune disease, most commonly myasthenia gravis, and rarely with autoimmune encephalitis. More recently, viral triggers including COVID‐19 have also been implicated in autoimmunity. We present a case of antibody‐positive autoimmune encephalitis that developed in the setting of COVID‐19 in a patient with thymomatous myasthenia gravis.

## Graphical abstract

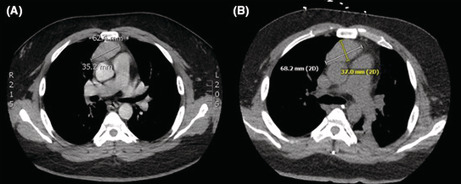

## 1 INTRODUCTION

Thymomas are commonly associated with paraneoplastic disorders due to their key role in adaptive immunity.^1^ The relationship between thymomas and myasthenia gravis (MG) is well established and bodes a relatively good prognosis for treatment response.^2^ The association between thymomas and autoimmune encephalitis is less well‐known, although prior literature suggests a poorer prognosis.^3^ More recently, the possibility that viral infections may contribute to the pathogenesis of autoimmune/paraneoplastic disorders in patients with coexisting thymomas has been raised, although the exact mechanism is still unclear.^4^

Here, we describe a case of α‐amino‐3‐hydroxy‐5‐methyl‐4‐isoxazolepropionic acid receptor (AMPA‐R) and collapsin response mediator protein 5 (CRMP‐5) antibody‐positive autoimmune encephalitis that developed in a patient with acetylcholine receptor (AChR)‐positive MG with a known thymoma in the setting of COVID‐19. While the presence of a thymoma is known to be associated with the development of autoimmune encephalitis, the temporal association with SARS‐CoV‐2 infection suggested that this may have triggered additional autoantibody production in our patient.

## 2 CASE REPORT

A 31‐year‐old man with AChR antibody‐confirmed MG presented to our emergency department with 3 weeks of progressively worsening confusion, forgetfulness, and visual hallucinations without insight into his deficits. He was noted to have dysphagia and dysarthria on the morning of presentation, prompting emergent evaluation. On examination, he was found to have symmetric, proximal muscle weakness involving all extremities and the neck. Initial negative inspiratory force (NIF) was measured at −20 cmH20.

**FIGURE 1:**
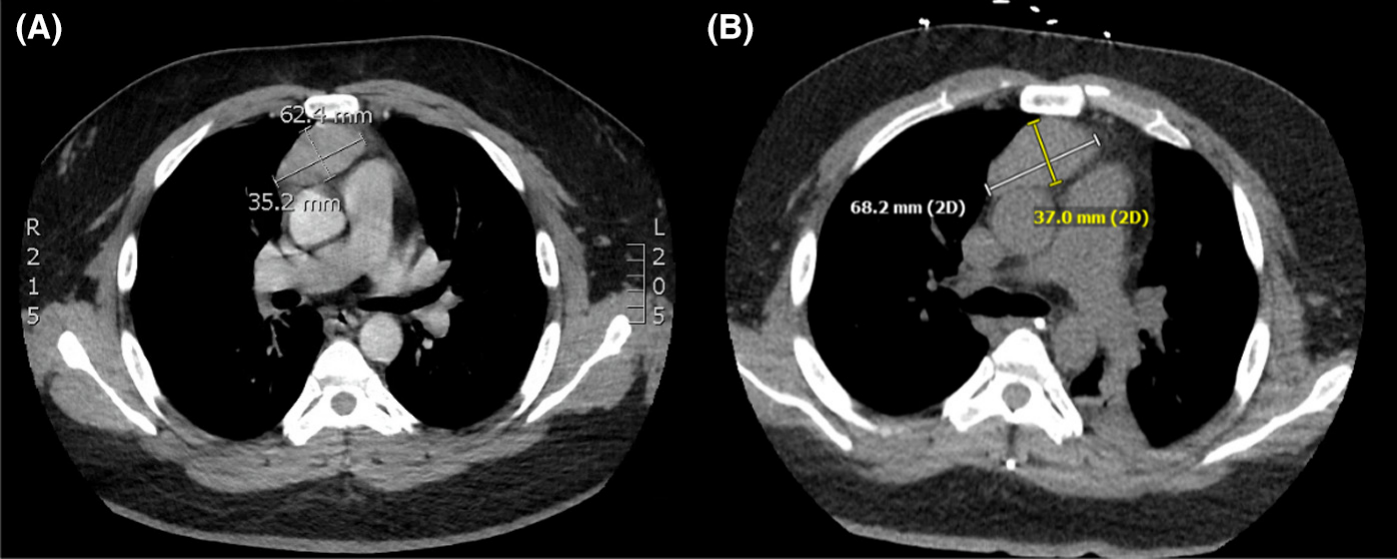
(A). Chest CT showing 6 × 3.5 cm anterior mediastinal mass consistent with thymoma 2 months prior to admission. (B). Chest CT showing anterior mediastinal mass which had increased in size to 3.7 × 6.8 cm.

The patient had been diagnosed with MG 3 months prior and was started on prednisone, pyridostigmine, and biweekly plasmapheresis. Given some residual weakness, eculizumab was added to his regimen 1 month earlier. Historical work‐up revealed a 6 × 3.5 cm thymoma (Figure 1), and he was already undergoing active evaluation for thymectomy as an outpatient. He had no known prior psychiatric history.

At the time of his admission, the initial medical work‐up revealed a positive SARS‐CoV‐2 PCR from nasopharyngeal swab and chest X‐ray showed bibasilar opacities consistent with multifocal pneumonia. He was admitted to the neurocritical care intensive care unit (NCCU) and electively intubated. Remdesivir and dexamethasone were started to treat any contribution of COVID‐19 to his respiratory distress. A superimposed myasthenic crisis was suspected, and intravenous immunoglobulin (IVIG) was administered.

However, given the change in mental status over 3 weeks and inconsistency of this finding with myasthenia alone, a broad neurologic work‐up was also initiated. Computed tomography (CT) and magnetic resonance imaging (MRI) of the brain obtained on hospital day (HD) 2 were notable only for incidental findings of mild inferior cerebellar tonsillar ectopia with an intact foramen magnum and a small gray matter heterotopia. Of note, MRI brain was repeated on HD 15 and HD 30 and remained stable, with the exception of development of possible mild central volume loss. Prolonged electroencephalogram (EEG) monitoring revealed moderate diffuse abnormalities consistent with encephalopathy, but no evidence of seizures or epileptiform discharges. Serum studies were remarkable for mildly elevated thyroid stimulating hormone (TSH) and thyroglobulin antibodies, as well as positive Ach‐R antibodies. Cerebrospinal fluid (CSF) analysis revealed a lymphocytic pleocytosis, with normal protein and glucose (Table 1). With the exception of positive SARS‐CoV‐2 PCR from nasopharyngeal swab, infectious work‐up was negative. Unfortunately, SARS‐CoV‐2 PCR testing on the CSF was unable to be performed by our laboratory at the time of the patient's admission. A systemic malignancy screen was otherwise unremarkable aside from the known thymoma, which had increased in size to 3.7 × 6.8 cm (Figure 1). Serial neurologic and respiratory examinations showed minimal improvement over the course of 2 weeks, and the patient remained unresponsive on sedation. The patient ultimately received 2 gm/kg of IVIG over 3 days and remained on eculizumab, remdesivir, and dexamethasone. Multiple attempts to wean the patient's ventilator requirements were unsuccessful, and the patient eventually underwent tracheostomy on HD 18. There were intermittent episodes of autonomic storming, medically managed with propranolol, clonidine, bromocriptine, morphine, and midazolam. He also developed dystonic posturing without EEG correlate.

**TABLE 1:** Summary of pertinent laboratory findings.

Serum studies		CSF studies		
			Tube #1	Tube #4
TSH IU/mL	5.94	RBC (mm^3)^	593	15
Thyroglobulin Ab	8.3	WBC (mm^3)^	21	12
Ach‐R‐binding Ab	10.2	Lymphocytes	90%	94%
Ach‐R‐modulating Ab	100%	Neutrophils	9%	3%
AMPA‐R Ab	Positive	Macrophages	1%	3%
CRMP‐5 IgG	Positive	Glucose	53	
P/Q Type Calcium Channel Ab	Positive (0.04)	Protein	37	
		Lactate	1.2	
		AMPA‐R Ab	Positive (1:256)	
		CRMP‐5 IgG	Positive (1:128)	

On HD 20, the patient was started on a 10‐session course of plasmapheresis. On HD 24, autoimmune serum (Mayo Clinic Laboratories, ENS2) and CSF (Mayo Clinic Laboratories, ENC2) panels returned positive for AMPA‐R antibodies (CSF titer 1:256) and CRMP‐5 antibodies (CSF titer 1:128) (Table 1). At this time, cardiothoracic surgery was consulted for thymectomy, which was carefully considered in multidisciplinary discussions due to the patient being critically ill in the ICU with poor neurologic function. The course of plasmapheresis ended on HD 39 and a robotic‐assisted thorascopic thymectomy was performed on HD 41 without acute complications. Neurological examination following procedure showed minimal improvement, and patient was noted to be nonverbal without ability to track, spontaneous movement, or withdrawal to noxious stimuli.

By HD 45, there continued to be little improvement in the patient's clinical status so rituximab was trialed. He received three weekly infusions of rituximab 375 mg/m2 while admitted (with the fourth planned for outpatient; however, he was briefly lost to follow‐up). On HD 60, the patient was deemed stable for discharge to a long‐term acute care hospital (LTACH). On discharge, he was only able to withdraw to noxious stimuli and had some spontaneous anti‐gravity movement in his bilateral upper extremities. He remained nonverbal with a tracheostomy in place.

The patient remained stable in the LTACH for approximately 1 month, until he was re‐admitted with pneumonia. During this hospitalization, he was treated with antibiotics and approximately 3 weeks after admission he self‐decannulated. Given increased respiratory strength at this point, his tracheostomy tube was not replaced and he gradually regained his speech and swallow function. By discharge, patient was noted to be conversing and responding.

The patient then followed up in neuroimmunology and neuromuscular clinics with gradual improvement in motor strength and cognition. He was maintained on mycophenolate 1000 mg twice daily and a prednisone taper. At his most recent follow‐up visit, approximately 16 months after his original presentation to the emergency department, he is almost completely off prednisone and has returned to his premorbid neurological function with plans to return to work.

## 3 DISCUSSION

Here, we present a case of a patient with pre‐existing thymoma and MG who developed AMPA‐R/CRMP‐5 receptor autoimmune encephalitis in the setting of SARS‐CoV‐2 infection. It is well established that thymomas are frequently associated with MG, occurring in approximately 15%–20% of cases.^1^ Less frequently, thymomas can be associated with other autoimmune disorders including various types of autoimmune encephalitides. In one retrospective cohort study of 43 patients with thymoma and autoimmune encephalitis, anti‐GABA_A_ receptor encephalitis was the most common, followed closely by AMPA‐R encephalitis.^5^ Notably, patients with AMPA‐R encephalitis had poorer outcomes, although this was likely due to more frequent co‐occurrence of onconeural antibodies such as CRMP‐5.^5^ In another case series, the prevalence of onconeural antibodies co‐occurring with AMPA‐R encephalitis was 32%.^3^ It has been shown that patients with a tumor but no onconeural antibodies had good survival, whereas when concomitant onconeural antibodies were present the majority of patients did not survive.^3^ The pathologic mechanism by which intracellular onconeural CRMP‐5 antibodies portend a poorer prognosis is likely due to CD8+ cytotoxic T‐cell‐mediated neuronal damage.^6^

Despite prior reports of poor prognosis, our case demonstrates that aggressive therapy with both immunotherapy and tumor resection should be pursued as there is a chance for survival and functional recovery. It should also be noted that there was a lag between time of treatment with thymectomy/immunotherapy and the onset of neurological recovery, but excellent supportive care allowed for this patient's improvement over time. This highlights that recovery from autoimmune encephalitis can be prolonged, but slow recovery is not a reason to withdraw care. Favorable prognosis was also demonstrated by another case series of five patients with co‐occurring anti‐AMPA‐R and anti‐CRMP5 receptor encephalitis, in which one out of five patients had a good recovery and returned to work. Similar to our patient, the single patient who recovered also had a thymoma and was treated with steroids, IVIG, Rituximab, and thymectomy.^7^

The relationships between viral infections, thymoma, and autoimmune disorders such as autoimmune encephalitis are becoming increasingly well‐recognized. Prior studies have implicated varicella‐zoster virus, herpes simplex virus, and Epstein–Barr virus as contributing pathogens to triggering thymoma‐associated autoimmune disorders,^4^ but we would like to highlight the first case of AMPA‐R and CRMP‐5 encephalitis associated with concurrent SARS‐CoV‐2 infection. COVID‐19 has commonly been associated with encephalopathy, but rarely seronegative autoimmune encephalitis has also been described.^8^, ^9^ There have also been case reports of COVID‐19‐associated anti‐NMDA‐R^10^, ^11^ and anti‐MOG encephalitis.^12^, ^13^ However, to our knowledge, this is the first case implicating COVID‐19 in the development of AMPA‐R/ CRMP‐5 autoimmune encephalitis.

This case is interesting and complex in that this patient had a thymoma and COVID‐19, both of which have been independently associated with autoimmune encephalitis. It is important to note that he presented with 3 weeks of progressive confusion, memory deficits, and weakness. He was admitted for myasthenic crisis. Given that he had no infectious symptoms (fever, cough, etc.), COVID‐19 was not initially suspected and was found on routine admission testing. He was electively intubated due to low NIF, but later developed radiographic findings consistent with COVID‐19 pneumonia. Given the onset of confusion and memory deficits 3 weeks prior and lack of infectious symptoms making it difficult to determine the exact onset of COVID‐19, we cannot rule out that autoimmune encephalitis started prior to SARS‐CoV‐2 infection. However, the temporal relationship with SARS‐CoV‐2 infection suggests that COVID‐19 in combination with the susceptibility to immune dysregulation from the underlying thymoma contributed to this patient's development of autoimmune encephalitis and that COVID‐19 precipitated his presentation in myasthenic crisis.

In summary, this is the first known case of AMPA‐R and CRMP‐5 antibody autoimmune encephalitis associated with SARS‐CoV‐2 infection. Furthermore, our case demonstrates that despite prior reports of a poor prognosis when there is co‐occurrence of CRMP5 antibodies, aggressive treatment with immunosuppression and cancer‐directed therapy can result in a dramatic recovery.

## AUTHOR CONTRIBUTIONS

**Jenelle Raynowska:** Writing – original draft; writing – review and editing. **Victoria Wu:** Writing – original draft; writing – review and editing. **Max Kazer:** Writing – review and editing. **Jamie LaBuzetta:** Supervision; writing – review and editing. **Dominic Ferrey:** Supervision; writing – review and editing. **Anastasie Dunn‐Pirio:** Supervision; writing – review and editing.

## FUNDING INFORMATION

None.

## CONFLICT OF INTEREST STATEMENT

The authors have no conflicts of interest to declare.

## ETHICS STATEMENT

Our patient provided consent for this case report. I think this correctly classifies as the ethics statement for our case report.

## Data Availability

Data sharing is not applicable to this article as no new data were created or analyzed in this study.
